# Oclacitinib Treatment and Surgical Management in a Case of Periocular Eosinophilic Furunculosis and Vasculitis with Secondary Eyelid Fusion in a Diabetic Cat

**DOI:** 10.3390/vetsci12060589

**Published:** 2025-06-15

**Authors:** Sarah Ehling, Anne Helene Marx, Claudia Busse, Andreas Beineke, Andrea Vanessa Volk

**Affiliations:** 1 Small Animal Hospital, Department of Dermatology, University of Veterinary Medicine Hannover Foundation, 30559 Hanover, Germany; sarah.ehling81@gmail.com (S.E.); anne.marx@tiho-hannover.de (A.H.M.); claudia.busse@tiho-hannover.de (C.B.); 2 Department of Pathology, University of Veterinary Medicine Hannover Foundation, 30559 Hanover, Germany; andreas.beineke@tiho-hannover.de

**Keywords:** eosinophilic furunculosis of the face, diabetes mellitus, oclacitinib

## Abstract

A 10-year-old male neutered British Shorthair cat with diabetes mellitus presented with an acute onset of unilateral swelling, erythema, alopecia and coalescing ulcerations of the face and periocular skin. Initial clinical differential diagnoses were trauma, infections (including feline respiratory viruses), arthropod bites, and eosinophilic dermatoses such as eosinophilic granuloma complex, mosquito-bite hypersensitivity and cutaneous adverse drug reaction. Histopathology revealed fulminant furunculosis with abundant eosinophils and vasculitis. Initial topical glucocorticoid treatment partially improved the clinical signs but severely raised serum glucose levels. As a result, systemic glucocorticoids and ciclosporin were not considered optimal treatments, and the off-label and short-term use of oclacitinib was chosen with the owner’s informed consent. This treatment induced fast remission of clinical signs with no recurrence for 17 months. Secondary fusion of the eyelids caused by cicatrization was surgically reconstructed to restore full function.

## Simple Summary

A 10-year-old male British Shorthair cat with diabetes mellitus was presented at a veterinary practice after suddenly developing swelling, redness, hair loss, and sores on one side of his face, especially around the eye. The vet first considered several possible causes, including an injury, parasites, infections (like cat flu), insect bites and allergic skin conditions. A skin sample showed the cat had a serious inflammation of the hair follicles, with a type of immune cell called eosinophils involved, as well as inflammation of the blood vessels. The vet first tried a steroid cream, which helped the skin but caused the cat’s blood sugar to rise—a problem for diabetic animals. Because of this, other strong anti-inflammatory medicines like steroid pills or ciclosporin were not good options. Instead, with the owner’s permission, the vet tried a different drug called oclacitinib (not usually used in cats). It worked very well, and the cat’s skin cleared up quickly, with no return of the problem for over a year. Later on, the cat needed surgery to fix his eyelids, which had stuck together as the skin healed, so he could blink normally again.

## 1. Background

Eosinophilic dermatoses are characterized by the influx of eosinophils into dermal tissues. In cats, these include eosinophilic granuloma complex (indolent ulcer, eosinophilic plaque and eosinophilic granuloma), miliary dermatitis and feline mosquito-bite hypersensitivity. These clinical presentations can be caused by a variety of factors but are most commonly thought to be the cutaneous manifestation of feline allergic disease, typically with a slow onset and chronic pruritus [1,2,3]. If ulcerations are present, feline viral infections, including upper respiratory viruses, should also be considered as differential diagnoses.

Clinically, acute eosinophilic dermatoses cause severe inflammation, pain and sometimes ulcerations. As a result, they require immediate local or systemic treatment with glucocorticoids [4]. When glucocorticoids are contraindicated, such as in case of co-morbidities, oclacitinib might be an equally fast-acting alternative. Oclacitinib, a JAK-1 inhibitor, reduces T_H_2-mediated immunity and levels of IL-5, an eosinophil-activating cytokine [5]. Oclacitinib is currently approved only for use in dogs with allergic or atopic dermatitis [6]. However, over the past 6–7 years, it has been used to treat alternative inflammatory diseases with surprisingly rapid results [7]. While oclacitinib is not licensed for cats, it has been used in isolated cases where glucocorticoids were unsuitable [8]. Because safety and efficacy data remain limited in cats, oclacitinib should only be considered when approved therapies fail or when concurrent medications contraindicate their use [7].

## 2. Case Presentation

A 10-year-old male neutered British Shorthair cat with diabetes mellitus presented with acute onset of severe pruritus, swelling and progressive alopecia on the right upper eyelid. The owner reported an initial red spot on the upper eyelid from which the lesions spread asymmetrically. The cat lived indoors in a multi-cat household and had no history of dermatological problems or pruritus. Co-morbidities included diabetes mellitus and feline odontoclastic resorptive lesions. At the time of referral, the cat was being treated with oral doxycycline (Doxybactin^®^, Dechra, Aulendorf, Germany) following a positive PCR result of *Mycoplasma felis* (throat swab). All other infectious organisms tested, including *Clamydia felis*, *calicivirus*, FHV-1 and *Bordetella bronchiseptica*, were negative. A corneal injury of the right eye as the initial cause of the blepharospasm and pruritus was ruled out by a negative fluorescein test. At this stage, the cat was already wearing a collar continuously to prevent scratching. Various consecutive treatments with topical (eye drops) and systemic glucocorticoids (over 48 h), antibiotics, and NSAIDs were ineffective in halting the progression of ulceration.

**Figure 1:**
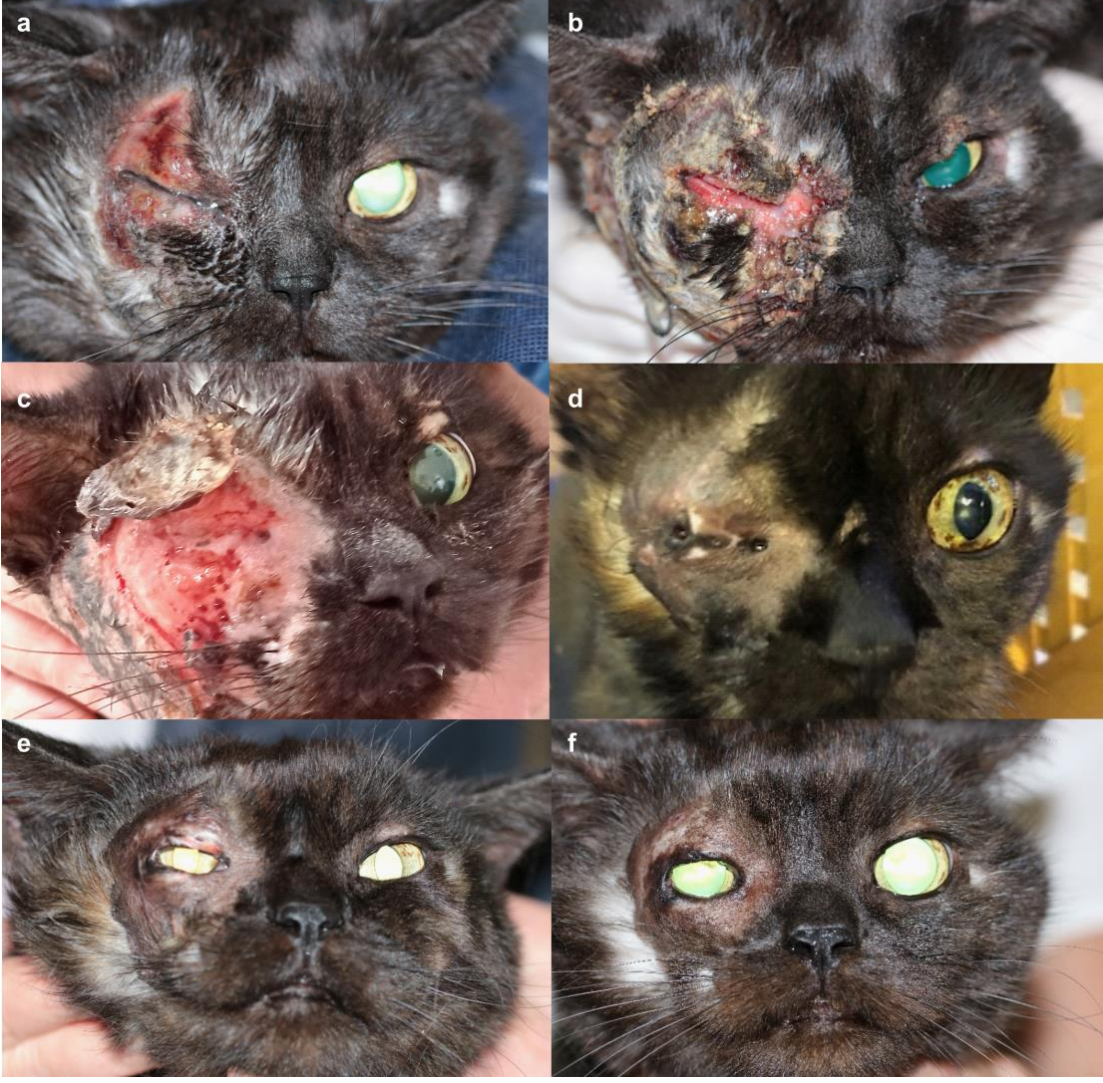
Timeline of progression of a ten-year old cat with suspected eosinophilic furunculosis. (**a**). Initial presentation three weeks post disease onset. (**b**). Four weeks. (**c**). Five weeks. (**d**). Three months. (**e**). Five days post ocular surgery. (**f**). Seventeen months post ocular surgery.

Three weeks after onset, the cat was referred to our ophthalmology and dermatology departments. Painful erosive-to-ulcerative lesions with marked erythema, alopecia and swelling were present on the upper and lower right eyelids. Satellite lesions of ulceration, erythema, and alopecia were observed on the ipsilateral pinna and neck with an asymmetrical distribution. The periocular skin was severely affected, leaving only a narrow area of the eyelid margin intact (Figure 1a). Swelling of the eyelids impaired the complete ophthalmic examination of the right eye. Tear production and intraocular pressure were within normal limits, and the corneal surface and intraocular structures were unremarkable as far as could be assessed. The fluorescein test was negative in both eyes. While awaiting biopsy under general anesthesia, the cat was treated with an NSAID (meloxicam, Metacam^®^, Boehringer Ingelheim, Ingelheim-Rhein, Germany) and doxycycline was continued. On the day of biopsy, skin ulcerations had progressed to involve the entire right eyelid margin. Extensive swelling rendered further ophthalmic examination of the right eye impossible, but transpalpebral ultrasonography confirmed an intact globe.

At this stage, clinical differential diagnoses for this acute, asymmetrical presentation included arthropod bites, infections (fungal, bacterial or viral, including feline respiratory viruses), trauma and cutaneous adverse drug reaction. Additional considerations included an atypical presentation of eosinophilic granuloma complex or mosquito-bite hypersensitivity.

During the initial consultation, ectoparasites were ruled out via hair plucking and coat brushing. Skin cytology of the most recent erosive lesions revealed small amounts of neutrophils without infectious agents. Blood chemistry and a complete blood count were unremarkable, and FIV-FeLV-tests were negative. The cat remained otherwise healthy, with a good appetite and no fever throughout the course of illness.

**Figure 2:**
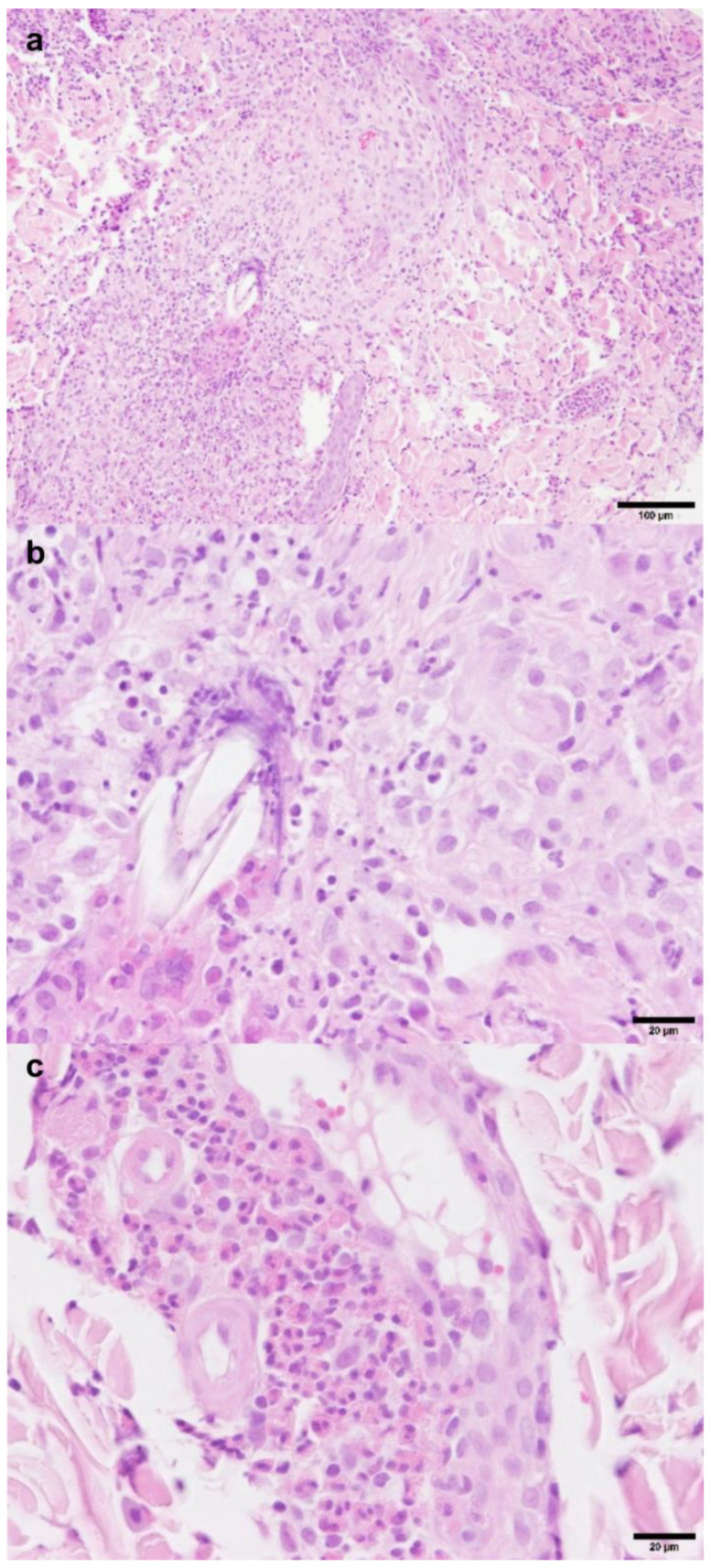
Histopathology H&E staining: eosinophilic furunculosis with secondary vasculitis. (**a**). Furunculosis with lymphohistiocytic inflammation and abundant eosinophils; (**b**,**c**). lymphohistiocytic inflammation with abundant eosinophils and vasculitis.

Histopathology of samples from the forehead, right eyelid and neck revealed severe, chronic, lymphohistiocytic folliculitis and furunculosis with abundant eosinophils and vasculitis (Figure 2). Neither parasitic nor infectious etiologies were identified, and macerated tissue culture failed to demonstrate infectious agents. Immunohistochemistry for feline herpesvirus and coronavirus antigens was negative, as was PAS staining.

After biopsy collection, initial topical treatment of the satellite lesions on the right neck and pinna with fusidic acid (to prevent bacterial infection) and betamethasone (Isaderm^®^, Dechra, Aulendorf, Germany) resulted in slight improvement. Unfortunately, topical glucocorticoid treatment had to be discontinued due to derailed serum glucose levels, which were monitored twice daily by the owner (Supplementary Figure S1). Periocular lesions progressed to full-thickness ulceration and necrosis by the fifth week after onset (Figure 1b,c).

Ciclosporin was not considered the next best choice in this case, primarily due to its slow onset of action and potential interference with serum glucose levels. After detailed discussion and consent from the owner, oclacitinib (Apoquel^®^, Zoetis Inc., Kalamazoo, MI, USA) was chosen for off-label, ideally short-term use at 1 mg/kg once daily [9]. The cat experienced mild gastrointestinal side effects (one instance of vomiting followed by a few days of diarrhea), leading to adjustment to 0.5 mg/kg twice daily. The cat tolerated this adjusted regimen well. Blood hematology was performed at the regular veterinarian and reported unremarkable. Blood glucose levels were monitored routinely by the owner twice daily, with no elevations reported (Supplementary Figure S1). The owner declined ectoparasite control treatment.

After three months, all skin lesions had resolved. Hypopigmented, alopecic scar tissue remained, leading to near-complete fusion of the eyelids and secondary visual impairment without signs of pruritus or pain (Figure 1d). Surgical intervention under general anesthesia was scheduled after the complete resolution of active inflammation. To restore eyelid function and vision, a full-thickness continuous incision of the scarred skin was performed, starting from the small remaining palpebral fissure opening nasally. Upon opening the fissure, adhesions of the palpebral conjunctivae of the third eyelid and the eyelids were removed. The wound margin of the upper eyelid was covered with palpebral conjunctiva, which was adapted using 6/0 Vicryl. A third eyelid flap was placed, while the lower eyelid wound was left to heal secondarily. Five days later, following removal of the flap, the patient was visual with a positive palpebral reflex. Eyelid motion was reduced, but complete closure was possible (Figure 1e). Oclacitinib was tapered in frequency over a one-month period (once daily for two weeks, every other day for two weeks) and then discontinued. Monthly rechecks alternated between hospital visits and phone consultations over the next 5 months, with a final consultation 17 months after surgery. During this whole time, no signs of recurrence of dermal disease were observed, and eyelid function as well as tear production remained stable throughout follow-up appointments (Figure 1f).

## 3. Discussion

This case report describes a cat with histopathologically confirmed eosinophilic furunculosis with vasculitis affecting the right periocular region of unknown cause. Therapy was complicated by diabetes mellitus, leading to an off-label use of oclacitinib, not intended to be life-long, if possible.

Among eosinophilic dermatoses in cats, a severe form of feline mosquito-bite hypersensitivity might be considered due to the comparable acute onset and ulceration with crusting on the face and pinna. In severe cases, histopathology reveals deep nodular eosinophilic dermatitis with folliculitis and furunculosis [10] (p. 452). One author attributes histopathological similarities with the canine folliculitis and furunculosis of the face [1]. In dogs, this disease presents peracutely with painful ulceration and crusts on the pinnae, lips or periocular regions. Etiologies in dogs include arachnids or insect bites, *Pelodera* species and hookworms [11]. The clinical presentation in the cat described here aligns with those associated with arthropod bites from spiders or ticks, which often affect the nose, ear tips and legs. Asymmetrical erythematous lesions may rapidly progress to necrosis with ulceration, crusting and alopecia [12]. The onset of this case occurred in December, and the cat lived exclusively indoors. While this makes a mosquito-triggered allergic reaction less likely, other bites (e.g., from spiders) can occur indoors throughout the year. Therefore, this case shares striking similarities with canine folliculitis and furunculosis of the nose, historically, clinically and histopathologically. Peripheral blood eosinophilia was not observed in this case, and it is also uncommon in canine cases [13].

Histopathology and microbiology excluded infectious differential diagnoses for furunculosis of the feline face. Among the remaining eosinophilic dermatoses in cats, eosinophilic granuloma complex and miliary dermatitis can have various causes but are most commonly considered cutaneous manifestations of feline allergic disease, which typically presents with slow onset and chronic pruritus. These conditions rarely cause full-thickness ulceration and vasculitis [10,14]. Additionally, due to their systemic nature, most cases exhibit bilateral symmetric distribution. The cat described here had an acute onset of unilateral lesions and had never shown pruritus or skin lesions before and after this episode (17 months follow-up). Consequently, these diseases were considered unlikely.

Histopathologically, the vasculitis in the case is suggested to be secondary to furunculosis and not the primary cause. Furthermore, a cutaneous adverse drug reaction was deemed unlikely due to the marked asymmetrical distribution of the lesions. Additionally, the cat had no dietary changes, environmental changes or new medications prior to the onset of symptoms.

On cytology, eosinophils are typically expected to emerge from draining fistulous tracts [10] (p. 452). However, in this case, only a few neutrophils but no eosinophils were found. This could be due to superficial sampling or poor staining quality for eosinophils. Interestingly, a case report of a novel presentation of eosinophilic granuloma complex also failed to detect eosinophils on impression cytology [15]. Another possibility is that neutrophils, arriving due to secondary infection, masked the presence of relevant cells. At the time of consultation, the cat was also being treated with doxycycline for a *Mycoplasma* infection, which could explain the absence of bacteria on cytology (and in macerated tissue culture).

Treatment options for eosinophil-driven dermatoses involve topical and systemic glucocorticoids. In diabetic cats, glucocorticoids must be used cautiously due to their potential to disrupt glucose metabolism [16,17], as was evident in this case. The same is reported when ciclosporin is added [18]. Additionally, depending on the disease, ciclosporin has a delayed onset of action (4–6 weeks) [19], making it less suitable for acute eosinophilic dermatitis.

Although oclacitinib is not currently licensed for cats, it was chosen for its rapid efficacy compared to licensed ciclosporin [5]. Additionally, no impact on blood glucose but minimal effect on fructosamine levels was reported in one study [20]. Short-term studies suggest that doses of 1–2 mg/kg up to twice daily for 28 days are generally safe and well-tolerated [9,20,21]. However, there has been a reported case of fatal toxoplasmosis following oclacitinib treatment at 1 mg/kg twice daily [22]. In the case presented here, the owner refused toxoplasmosis testing because the cat was a strict indoor cat and never fed raw meat.

Severe inflammation resulting in loss of normal eyelid tissue can cause eyelid deformities and malpositioning (e.g., entropion, ectropion and lagophthalmos), potentially leading to ocular surface disease and vision impairment [23] (p. 2752). In the presented case, inflammation and ulceration were extensive, leading to near-complete loss of eyelid margin and fusion of the palpebral fissure, resulting in unilateral vision loss. During active inflammation, the ocular surface was managed with mucinomimetic and lacrimomimetic therapy to prevent corneal disease. Potential complications associated with the sharp dissection required to reopen the fused palpebral fissure included lagophthalmos, possibly due to an unpredictable loss of the orbicularis oculi muscle [24], qualitative dry eye disease due to destruction of the Meibomian glands [25] and repeated refusion of the palpebral fissure due to the absence of intact eyelid margins. However, none of these complications occurred in this case. The surgical procedure successfully alleviated pain, restored vision and eyelid motion and enabled complete eyelid closure in the affected eye.

In summary, none of the differential diagnoses share the fulminant, rapid clinical onset combined with the histopathological diagnosis of eosinophilic furunculosis with vasculitis of the face, as seen in this case.

## 4. Conclusions

This report described a case of aggressive eosinophilic furunculosis with vasculitis affecting the periocular skin of a diabetic cat, which led to the destruction of the eyelids and subsequent fusion of the palpebral fissure. Treatment with oclacitinib resulted in complete healing within three months. Subsequent surgical dissection and reconstruction of the eyelid fully restored vision and largely restored eyelid function. No relapse occurred during 17 months of follow-up. The use of oclacitinib in cats should be carefully considered, particularly in diabetic cases where licensed products may exacerbate the condition. The potential for life-threatening side effects must be thoroughly discussed with owners before initiating treatment.

## Data Availability

The original contributions presented in this clinical case are included in the article/Supplementary Materials. Further inquiries can be directed to the corresponding author.

## Supplementary Materials

The following supporting information can be downloaded at: https://www.mdpi.com/article/10.3390/vetsci12060589/s1, Figure S1: Total insulin dose per day from three weeks post clinical onset to end of treatment.

## References

[1] Bloom P.B. (2006). Canine and feline eosinophilic skin diseases. Vet. Clin. N. Am. Small Anim. Pract..

[2] Forsythe P. (2011). Feline eosinophilic dermatoses Part 2: Further investigation and long-term management. Companion Anim..

[3] Forsythe P. (2011). Feline eosinophilic dermatoses Part 1: Aetiology, clinical signs and investigation. Companion Anim..

[4] Kouro T., Takatsu K. (2009). IL-5- and eosinophil-mediated inflammation: From discovery to therapy. Int. Immunol..

[5] Jasiecka-Mikolajczyk A., Jaroszewski J.J., Maslanka T. (2021). Oclacitinib, a Janus Kinase Inhibitor, Reduces the Frequency of IL-4- and IL-10-, but Not IFN-gamma-, Producing Murine CD4(+) and CD8(+) T Cells and Counteracts the Induction of Type 1 Regulatory T Cells. Molecules.

[6] Gonzales A.J., Bowman J.W., Fici G.J., Zhang M., Mann D.W., Mitton-Fry M. (2014). Oclacitinib (APOQUEL((R))) is a novel Janus kinase inhibitor with activity against cytokines involved in allergy. J. Vet. Pharmacol. Ther..

[7] Marsella R., Doerr K., Gonzales A., Rosenkrantz W., Schissler J., White A. (2023). Oclacitinib 10 years later: Lessons learned and directions for the future. J. Am. Vet. Med. Assoc..

[8] Carrasco I., Martinez M., Albinyana G. (2021). Beneficial effect of oclacitinib in a case of feline pemphigus foliaceus. Vet. Dermatol..

[9] Ferrer L., Carrasco I., Cristofol C., Puigdemont A. (2020). A pharmacokinetic study of oclacitinib maleate in six cats. Vet. Dermatol..

[10] Gross T.L., Ihrke P.J., Walder E.J., Affolter V.K. (2005). Skin Diseases of the Dog and Cat: Clinical and Histopathologic Diagnosis.

[11] Pouleur-Larrat B., Fantini O., Pin D. (2017). Dermatitis due to Pelodera strongyloides with eosinophilic folliculitis-furunculosis in 2 young dogs. Summa Anim. Compagnia.

[12] Bevier D.E. (1999). Insect and arachnid hypersensitivity. Vet. Clin. N. Am. Small Anim. Pract..

[13] Guaguere E. (1996). Topical treatment of canine and feline pyoderma. Vet. Dermatol..

[14] Porcellato I., Giontella A., Mechelli L., Del Rossi E., Brachelente C. (2014). Feline eosinophilic dermatoses: A retrospective immunohistochemical and ultrastructural study of extracellular matrix remodelling. Vet. Dermatol..

[15] Hopke K.P., Sargent S.J. (2019). Novel presentation of eosinophilic granuloma complex in a cat. JFMS Open Rep..

[16] McCann T.M., Simpson K.E., Shaw D.J., Butt J.A., Gunn-Moore D.A. (2007). Feline diabetes mellitus in the UK: The prevalence within an insured cat population and a questionnaire-based putative risk factor analysis. J. Feline Med. Surg..

[17] Nerhagen S., Moberg H.L., Boge G.S., Glanemann B. (2021). Prednisolone-induced diabetes mellitus in the cat: A historical cohort. J. Feline Med. Surg..

[18] Case J.B., Kyles A.E., Nelson R.W., Aronson L., Kass P.H., Klose T.C., Bailiff N.L., Gregory C.R. (2007). Incidence of and risk factors for diabetes mellitus in cats that have undergone renal transplantation: 187 cases (1986–2005). J. Am. Vet. Med. Assoc..

[19] Noli C., Scarampella F. (2006). Prospective open pilot study on the use of ciclosporin for feline allergic skin disease. J. Small Anim. Pract..

[20] Carrasco I., Ferrer L., Puigdemont A. (2022). Efficacy of oclacitinib for the control of feline atopic skin syndrome: Correlating plasma concentrations with clinical response. J. Feline Med. Surg..

[21] Lopes N.L., Campos D.R., Machado M.A., Alves M.S.R., de Souza M.S.G., da Veiga C.C.P., Merlo A., Scott F.B., Fernandes J.I. (2019). A blinded, randomized, placebo-controlled trial of the safety of oclacitinib in cats. BMC Vet. Res..

[22] Moore A., Burrows A.K., Malik R., Ghubash R.M., Last R.D., Remaj B. (2022). Fatal disseminated toxoplasmosis in a feline immunodeficiency virus-positive cat receiving oclacitinib for feline atopic skin syndrome. Vet. Dermatol..

[23] Gelatt K.N., Ben-Shlomo G., Gilger B.C., Hendrix D.V.H., Kern T.J., Plummer C.E. (2021). Veterinary Ophthalmology.

[24] Pereira M.V., Gloria A.L. (2010). Lagophthalmos. Semin. Ophthalmol..

[25] Al-Dossy S.K. (2023). Correlation between Ocular Surface Parameters and the Severity of Blepharitis in Patients with Dry Eye. Pak. Heart J..

