# Supplementary material for: Oclacitinib Treatment and Surgical Management in a Case of Periocular Eosinophilic Furunculosis and Vasculitis with Secondary Eyelid Fusion in a Diabetic Cat
Source: Vet Sci. 2025 Jun 15;12(6):589. doi: 10.3390/vetsci12060589 (PMC12197539; doi:10.3390/vetsci12060589)
Supplement: Supplementary file 1 [file vetsci-12-00589-s001.zip › vetsci-3656985-supplementary.pdf]

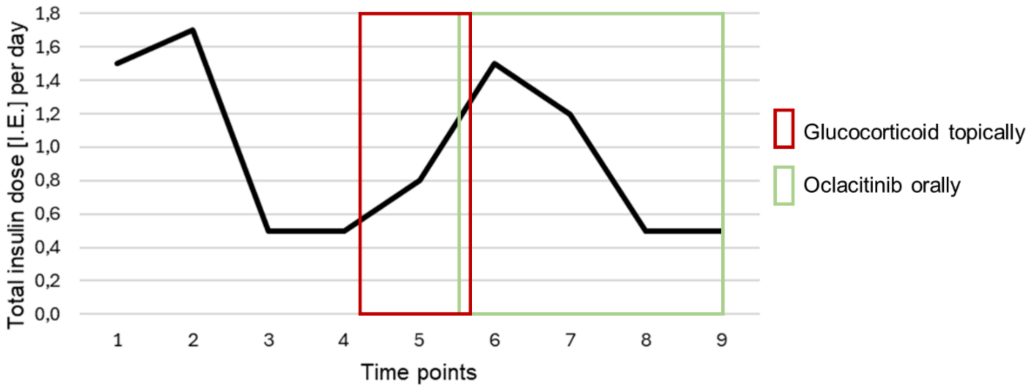

Supplementary Figure S1. Total insulin dose per day from three weeks post clinical onset to end of treatment
